# The Early Diagnosis of Epithelioma

**Published:** 1891-11

**Authors:** William S. Gottheil

**Affiliations:** Lecturer on Dermatology, New York Polyclinic; 25 West 53d Street, New York City


### Southern Medical Record.

A MONHLY JOURNAL OF MEDICINE AND SURGERY.

VOL. XXI. ATLANTA, GA., NOVEMBER, 1891. No. 11

### Original Articles.

### THE EARLY DIAGNOSIS OF EPITHELIOMA.

BY WILLIAM S. GOTTHEIL, M. D.,
Lecturer on Dermatology, New York Polyclinic.

Epithelioma, in its earlier stages, is frequently neglected by both physician and patient. This is unfortunate, but not strange. Who would suspect the innocent-looking little wart that has remained quiescent for so many years of containing within itself possibilities so portentous? It cannot be that this senile mole, hurtful till now only by ugliness, will suddenly blossom out into that evil plant we know and dread as cancer. It seems impossible that from structures so simple and so innocent such a new growth can arise.

Yet such is the case. Almost every patient will tell this story: "Why, doctor, I had a little wart there ever since I can remember, and it never gave me any trouble; but two years ago it began to itch a little, and I scratched it; then little scabs formed, which I removed as fast as they came; then the wart began to grow, and it has continued to grow up to the present time." So well recognized is this relationship between innocent hyperplasias and malignant new growths, that some surgeons have advocated the systematic removal of all warts and excrescences from the faces and hands of elderly people as a preventive measure.

It is of small moment what theory we adopt as to the cause that suddenly stimulates into vigorous and irregular life a group of epithelial cells that have remained lazy and quiescent for perhaps half a century. If we hold to the pathology of the schools, we may, with Cohnheim, talk of embryonic cells "left over;" if we are more radical, we can turn to the as yet undiscovered germ which is supposed by some to be the etiological factor. It is sufficient for us to know that the wart has become an epithelioma; that the epithelial cells have begun to proliferate rapidly; that a morbid process has started which, unless interfered with, will end only with the life of the individual affected.

But *that* fact we must know, and know early. It will make all the difference to us between a small and easily removable morbid growth, and a destructive process so extensive that all our efforts to stay its progress may be in vain. It will make all the difference to the patient between a small and easily-cured defect, and a hideous malady that requires severe operative procedures, and may destroy organs and life. It is the main purpose of this paper to insist upon this point, and show the data that enable us to make an early diagnosis. For it is quite safe to say that every epithelioma, recognized early and treated radically, can be easily and thoroughly cured.

Unfortunately, but too often, the patient fails to appreciate the importance of the little irritable wart in its early stages; and unfortunately, also, the physician very frequently does not recognize its nature until it has acquired considerable extent.

And so the little tumor is allowed to grow and spread—slowly and imperceptibly—until in the course of months and years the patient becomes alarmed and the physician's attention is awakened.

It is strange, but nevertheless true, that in the majority of cases the treatment used before is signally inappropriate. We are dealing with a soft and rapidly growing tissue of but little resisting power; the problem is to remove it radically. Our patient is old, perhaps, and dreads the knife, so the surgeon resorts to other methods. He applies carbolic acid, perhaps, or nitrate of silver, or bluestone. Week by week the "caustic" is applied, and week by week the epithelioma seems to thrive upon it and grow. It spreads and involves neighboring parts—the eyelids, the lips, the alæ nasi. This at once complicates matters, and renders the case much more difficult to treat. Disheartened at the non-result of treatment, the patient tries other doctors—including the quacks—or he takes cuticura—

that mysterious and money-making remedy that is "good" for all skin diseases. Finally, if he is lucky, and the disease has not progressed too far, he falls in the hands of one who knows what to do for him, and is cured at the expense of great disfiguring scars, destroyed eyes, deformed nose and mouth. Or, again, he may not be so lucky, and the last months of his life may be spent in the endeavor to stay the ravages made by the disease in vital organs.

This is no overdrawn picture; it is a true account of what takes place every day. Here, for instance, is the story of a merchant from a large Southern city, who came to me a few days ago with an epithelioma of six years standing. It is nearly three inches long by one inch broad, situated on the left side of the forehead, and involving the eyebrow. "I was treated four years ago for two years by my family physician; he used acids of various kinds very often, but did not succeed in eradicating it. I then used bluestone on it for two years, but without result. It has steadily increased in size all the time."

How, then, shall we recognize these growths early, and what are the best means for us to employ for their eradication?

There is no difficulty in the recognition of a fully developed case; but with those in the earlier stages a close examination is necessary. Not every wart is a cancer, though it may become one. Still, it is better to err on the side of caution than the reverse. Removal is easy, safe and painless in these early stages. It is a safe rule to treat every irritated or suspicious excrescence on the face of a person over fifty years of age as an epithelioma.

We have at first an irritated wart—either of the ordinary kind, or that flatter, larger and pigmented variety so common on the faces of the aged, known as verrucca senilis. The patient has noticed, perhaps, that it is getting a little larger lately. It irritates him, or it itches, and he has scratched it. A thin, small scab lies on its surface; he removes it, and it returns again.

If, now, we examine this flattened wart very closely, we shall probably notice that there is a distinct very narrow, rounded and swollen margin to it. If we look at this margin by an oblique light, we notice that it has a whitish "glance"—just as if a very small band of spermaceti had been let into the skin just below the surface epithelium. Over this waxy margin a few very fine but distinct red vessels may be coursing.

The faintest trace of slightly elevated waxy margin suffices for a diagnosis. Nothing but an epithelium shows this peculiarity. The waxy appearance is due to the accumulation of masses of proliferated epithelial cells, and the dilated vessels coursing over it are simply distended superficial capillaries.

As the tumor grows these characters become more marked The waxy margin enlarges, the number of capillaries increases. Should the center of the mass of epithelia break down from growth in excess of vascular supply possibilities, the margins of the ulcer will show the diagnostic characters. Should it take on the fungus form, which is so common, at the base of the cauliflower moss, the skin will show it.

Other factors may help us and complete the diagnosis; but the symptoms above mentioned are perfectly characteristic.

And now a brief consideration of those maladies that simulate epithelioma sufficiently to be mistaken for it; they are not numerous, nor is their differential diagnosis difficult.

As I have mentioned above, epithelioma frequently begins as a development of an ordinary wart, or as a structure so similar to one as to be undistinguishable from it. But the ordinary venucea is usually multiple, is commonest upon the hands, and is found in youthful individuals. The epitheliomatous wart is generally single, is situated in a vast majority of cases on the face, and affects individuals almost always over forty years of age. Great reliance can be placed upon the presence or absence of subjective symptoms. Warts are painless, do not itch, and are unnoticeable, save as a deformity. Epitheliomata are always irritable; they itch or burn; the patient scratches off the top, which is then covered by a scab. The flat, blackish venucea senilis is distinguished in exactly the same way from the malignant growth. Usually, within a few weeks of the onset of these subjective symptoms, the characteristic appearances of an epithelioma recounted above have appeared, and the diagnosis is no longer a matter of doubt.

But it is with a tubercular or ulcerative syphiloderm that epithelioma is most likely to be confounded. In all its stages it may resemble one or other of the many forms of this specific disease. As regards the tubercular or syphiloderm, the following points may be noted: it is usually multiple or pronged; it is not more prone to affect the face than other portions of the surface; it has lasted a short time only—a few weeks; it occurs at any age, and other evidences of past or present syphilis may be found. The epithelioma, on the other hand, is more often single—or if multiple there is no circinate grouping; its most frequent seat is on the face, and especially in the neighborhood of the mucous orifices; its history is a matter of months or years; it almost always occurs in elderly people; it is entirely local and unconnected with other diseases.

When the syphiloderm has ulcerated, the diagnosis is more difficult. Here again the patient's age and the history of the lesion will help us; but the main point is that the margins of the ulcer in either case presents the characters of the original tubercle from which it developed. The margins of the syphilitic ulcers are firm and moderately infiltrated; its edges are undermined; its base is sloughy. The margins of the cancerous ulcers are hard and have the peculiar waxily-infiltrated appearance mentioned above; minute blood-vessels course over it; its base is bright red, bleeds easily, and may be filled with hypertrophic fungus granulations.

It does not seem possible that there can be any difficulty in diagnosing epithelioma from lupus vulgaris. Lupus, as a rule, has lasted for many years; it begins in adolescent life; it heals in some parts with superficial scars, and spreads in others; it tends to close up any natural orifice that it invades, and the characteristic soft brownish nodules are found in the scar and in the margins of the ulcer. These points are characteristic enough to prevent any mistake.

Epithelioma, then, we may say, can be diagnosed, and ought to be diagnosed, in its earlier stages; its radical treatment is then easy and successful. Nevertheless, those who have the clinical opportunity of seeing many of these cases, know how very frequently it is not recognized.

*25 West 53d Street, New York City.*

